# Experimental Infection of Mexican Free-Tailed Bats (*Tadarida brasiliensis*) with SARS-CoV-2

**DOI:** 10.1128/msphere.00263-22

**Published:** 2023-01-04

**Authors:** J. S. Hall, E. Hofmeister, H. S. Ip, S. W. Nashold, A. E. Leon, C. M. Malavé, E. A. Falendysz, T. E. Rocke, M. Carossino, U. Balasuriya, S. Knowles

**Affiliations:** a U.S. Geological Survey, National Wildlife Health Center, Madison, Wisconsin, USA; b Louisiana Animal Disease Diagnostic Laboratory, School of Veterinary Health Medicine, Louisiana State University, Baton Rouge, Louisiana, USA; c Department of Pathobiological Sciences, School of Veterinary Health Medicine, Louisiana State University, Baton Rouge, Louisiana, USA; Icahn School of Medicine at Mount Sinai

**Keywords:** bats, SARS-CoV-2, susceptibility, infection

## Abstract

The severe acute respiratory syndrome coronavirus-2 (SARS-CoV-2) virus is thought to have originated in wild bats from Asia, and as the resulting pandemic continues into its third year, concerns have been raised that the virus will expand its host range and infect North American wildlife species, including bats. Mexican free-tailed bats (Tadarida brasiliensis) live in large colonies in the southern United States, often in urban areas and, as such, could be exposed to the virus from infected humans. We experimentally challenged wild T. brasiliensis with SARS-CoV-2 to determine the susceptibility, reservoir potential, and population impacts of infection in this species. Of 10 bats oronasally inoculated with SARS-CoV-2, 5 became infected and orally excreted moderate amounts of virus for up to 18 days postinoculation. These five subjects all seroconverted and cleared the virus before the end of the study with no obvious clinical signs of disease. We additionally found no evidence of viral transmission to uninoculated subjects. These results indicate that while T. brasiliensis are susceptible to SARS-CoV-2 infection, infection of wild populations of T. brasiliensis would not likely cause mortality. However, the transmission of SARS-CoV-2 from T. brasiliensis to or from humans, or to other animal species, is a possibility requiring further investigation to better define.

**IMPORTANCE** As the COVID-19 pandemic has continued for 3+ years, there has been increasing concern that the SARS-CoV-2 virus will enter wildlife populations and potentially create new reservoirs where the virus could adapt to a new host and create variants. This is particularly possible with species that reside in man-made structures, in proximity to infected human populations. Mexican free-tailed bats (Tadarida brasiliensis) live in large colonies, often in urban settings and, thus, can be exposed by infected humans and potentially transmit the virus to new hosts. We experimentally challenged T. brasiliensis with SARS-CoV-2 and revealed that they are susceptible to the virus and excrete moderate amounts for up to 18 days postinoculation. This is important information for wildlife biologists, wildlife rehabilitation workers, and the general public that may contact these animals.

## INTRODUCTION

As we enter the third year of the severe acute respiratory syndrome coronavirus-2 (SARS-CoV-2) pandemic, many unanswered questions remain regarding the ecology of the virus. How the virus interacts with wild species is critical knowledge to obtain, including whether (i) North American wildlife can act as reservoirs of the virus, (ii) the virus can adapt genetically to new hosts and become more virulent, and (iii) the virus can affect the health of wild populations, particularly threatened or endangered species.

SARS-CoV-2 naturally infects several wild species, including captive wild animals. For example, American minks (Neovison vison) were infected with SARS-CoV-2 in the United States in proximity to domestic mink farm operations (1). Antibodies to SARS-CoV-2 were detected in white-tailed deer (Odocoileus virginianus) indicating exposure to the virus (2), and infection was later confirmed in this species (3). Captive wild animals in zoos, particularly members of the *Felidae* family, were infected with the virus (4), and several North American species have experimentally been shown susceptible to the virus, including deer mice (Peromyscus maniculatus), striped skunks (Mephitis mephitis), and bushy-tailed woodrats (Neotoma cinerea) (5).

Because evidence indicates that SARS-CoV-2 possibly originated in Asian bats (6–8), concern that the virus could infect North American bat species has been raised, particularly for bat populations under severe threat from another pathogen, Pseudogymnoascus destructans (9, 10). Whether North American bats could provide a reservoir of the virus and additional routes of transmission to humans and other susceptible species, as well as any effects of SARS-CoV-2 on populations, is important to determine, as is any management measures that could be used to help protect these populations.

Big brown bats (Eptesicus fuscus) were previously challenged with SARS-CoV-2 and demonstrated resistance to infection (11). This species often encounters humans, as they frequently reside in anthropogenic structures, including occupied homes and other buildings. Another common North American bat species, the Mexican free-tailed bat (Tadarida brasiliensis), resides in very large colonies in the southern United States, often in urban areas. This species is migratory and, if susceptible to SARS-CoV-2, could transport the virus to/from Central and South America on their migratory routes. In this study, we challenged T. brasiliensis with SARS-CoV-2 to determine their susceptibility to infection, reservoir potential, the adaptability of the virus to a new potential host, and potential effects of the virus on their populations.

## RESULTS

### Presence of coronaviruses in Mexican free-tailed bats before inoculation.

RT-PCR analyses of fecal material collected from the T. brasiliensis before virus challenge revealed no evidence of alpha- or betacoronavirus infection, in any subject (Table S1 in the supplemental material).

10.1128/msphere.00263-22.1 TABLE S1 Rabies diagnostic results from Mexican free-tailed bats by direct fluorescent antibody (DFA) and the presence of alpha- or betacoronaviruses prior to initiation of the SARS-CoV-2 challenge study. *^1^*a, adult; j, juvenile. ^2^Pos, rabies virus presence in brain tissue; Neg, rabies virus absent in brain tissue or coronavirus absent in fecal material. ^3^None, bat was euthanized before SARS-CoV-2 challenge. Download Table S1, DOCX file, 0.01 MB. Copyright © 2023 Hall et al. 2023 Hall et al. https://creativecommons.org/licenses/by/4.0/ This is an open-access article distributed under the terms of the Creative Commons Attribution 4.0 International license.

### Rabies virus infection.

Before SARS-CoV-2 inoculation, one bat exhibited loss of appetite, aggressive behavior toward its cagemate, and weight loss over 3 to 4 days. We euthanized this bat and tested it for rabies. It was positive for the presence of rabies virus by direct fluorescent antibody test (DFA); therefore, we tested all the remaining T. brasiliensis after euthanasia and all were negative for the presence of rabies virus (Table S1).

### SARS-CoV-2 excretion after experimental inoculation.

Quantitative reverse transcription-PCR (qRT-PCR) analysis of oral swabs is shown in Table 1. Of the 10 SARS-CoV-2 inoculated T. brasiliensis, five (bats 104, 110, 118, 124, and 123) excreted detectable viral RNA. Two additional bats (111, 129) had high cycle threshold (C_t_) readings only on the first sampling after inoculation (DPI 2) and likely was detection of residual inoculum. The duration of oral excretion ranged from DPI 6 (bat 123) to 18 DPI (Bat 118). The maximum amount of virus detected on an oral swab was 4.82 × 10^3^ TCID_50_ equivalent/mL (bat 118 DPI 4). In contrast to oral excretion, no virus was detected in rectal swabs from any T. brasiliensis (Table 2). The effect of the bat’s age on susceptibility was inconclusive as four of the infected bats were adults and one was a juvenile, but the numbers were too small to be reliable.

**TABLE 1:** Quantitative RT-PCR analyses of oral swabs from Mexican free-tailed bats inoculated with SARS-CoV-2

Bat ID	Treatment^*a*^	Age^*b*^	DPI^*c*^ 0	DPI 2	DPI 4	DPI 6	DPI 8	DPI 10	DPI 12	DPI 14	DPI 16	DPI 18	DPI 20
102	Control	j	No Ct^*d*^	No Ct	No Ct	No Ct	No Ct	No Ct	No Ct	No Ct	No Ct	No Ct	No Ct
108	Control	j	No Ct	No Ct	No Ct	No Ct	No Ct	No Ct	No Ct	No Ct	No Ct	No Ct	No Ct
104	Inoc.	a	No Ct	**7.53**	**2.8 × 10** ^1^	**2.32**	**2.15**	**1.97**	No Ct	No Ct	X^*e*^	x	x
103	Contact	j	No Ct	No Ct	No Ct	No Ct	No Ct	No Ct	No Ct	No Ct	x	x	x
112	Inoc.	j	No Ct	No Ct	No Ct	No Ct	No Ct	No Ct	x	x	x	x	x
113	Contact	a	No Ct	No Ct	No Ct	No Ct	No Ct	No Ct	No Ct	No Ct	No Ct	No Ct	No Ct
110	Inoc.	a	No Ct	**2.96 × 10** ^3^	**3.14 × 10** ^3^	**2.51 × 10** ^2^	**1.07 × 10** ^3^	**3.10 × 10** ^2^	**9.08 × 10** ^1^	**3.44**	**1.37 × 10** ^1^	No Ct	No Ct
117	Contact	a	No Ct	No Ct	No Ct	No Ct	No Ct	No Ct	No Ct	No Ct	No Ct	No Ct	No Ct
118	Inoc.	a	No Ct	**8.63 × 10** ^2^	**4.82 × 10** ^3^	**1.82 × 10** ^3^	**2.18 × 10** ^3^	**5.99 × 10** ^1^	**4.74 × 10** ^1^	**2.08 × 10** ^2^	**4.74 × 10** ^2^	**4.30 × 10** ^1^	No Ct
114	Contact	j	No Ct	No Ct	No Ct	No Ct	No Ct	No Ct	No Ct	No Ct	No Ct	No Ct	No Ct
120	Inoc.	j	No Ct	No Ct	No Ct	No Ct	No Ct	No Ct	No Ct	No Ct	No Ct	No Ct	No Ct
121	Contact.	j	No Ct	No Ct	No Ct	No Ct	No Ct	No Ct	No Ct	No Ct	No Ct	No Ct	No Ct
124	Inoc.	j	No Ct	**6.80 × 10** ^2^	**2.52 × 10** ^2^	**6.56 × 10** ^2^	**1.00 × 10** ^3^	**2.18 × 10** ^1^	No Ct	No Ct	No Ct	No Ct	No Ct
125	Contact	a	No Ct	No Ct	No Ct	No Ct	No Ct	No Ct	No Ct	No Ct	No Ct	No Ct	No Ct
111	Inoc.	j	No Ct	**6.24**	No Ct	No Ct	No Ct	No Ct	No Ct	No Ct	No Ct	No Ct	No Ct
109	Contact	j	No Ct	No Ct	No Ct	No Ct	No Ct	No Ct	No Ct	No Ct	No Ct	No Ct	No Ct
123	Inoc.	a	No Ct	**1.94 × 10** ^2^	**1.39 × 10** ^2^	**6.00 × 10** ^1^	No Ct	No Ct	No Ct	No Ct	No Ct	No Ct	No Ct
122	Contact	j	No Ct	No Ct	No Ct	No Ct	No Ct	No Ct	No Ct	No Ct	No Ct	No Ct	No Ct
127	Inoc.	a	No Ct	No Ct	No Ct	No Ct	x	x	x	x	x	x	x
128	Contact	a	No Ct	No Ct	No Ct	No Ct	x	x	x	x	x	x	x
129	Inoc.	j	No Ct	**1.38 × 10** ^1^	No Ct	No Ct	No Ct	No Ct	No Ct	No Ct	No Ct	No Ct	No Ct

a Inoc, bat oronasally inoculated with 10^5^TCID_50_ SARS-CoV-2; Contact, uninoculated bat cohoused with inoculated bat to determine virus transmission. The SARS-CoV-2 positive swab samples are highlighted in bold.

b a, adult; j, juvenile.

c Day postinoculation.

d No Ct, no detectable real-time PCR amplification. Quantities of virus based on standard curve of known virus concentrations and expressed as TCID_50_ equivalent/mL.

e x, Bat euthanized after previous sampling DPI.

**TABLE 2:** Quantitative RT-PCR analyses of rectal swabs from Mexican free-tailed bats inoculated with SARS-CoV-2

Bat ID	Treatment^*a*^	DPI^*b*^ 0	DPI 2	DPI 4	DPI 6	DPI 8	DPI 10	DPI 12	DPI 14	DPI 16	DPI 18	DPI 20
102	Control	No Ct^*c*^	No Ct	No Ct	No Ct	No Ct	No Ct	No Ct	No Ct	No Ct	No Ct	No Ct
108	Control	No Ct	No Ct	No Ct	No Ct	No Ct	No Ct	No Ct	No Ct	No Ct	No Ct	No Ct
104	Inoc.	No Ct	No Ct	No Ct	No Ct	No Ct	No Ct	No Ct	No Ct	X^*d*^	x	x
103	Contact.	No Ct	No Ct	No Ct	No Ct	No Ct	No Ct	No Ct	No Ct	x	x	x
112	Inoc.	No Ct	No Ct	No Ct	No Ct	No Ct	No Ct	x	x	x	x	x
113	Contact	No Ct	No Ct	No Ct	No Ct	No Ct	No Ct	No Ct	No Ct	No Ct	No Ct	No Ct
110	Inoc.	No Ct	No Ct	No Ct	No Ct	No Ct	No Ct	No Ct	No Ct	No Ct	No Ct	No Ct
117	Contact	No Ct	No Ct	No Ct	No Ct	No Ct	No Ct	No Ct	No Ct	No Ct	No Ct	No Ct
118	Inoc.	No Ct	No Ct	No Ct	No Ct	No Ct	No Ct	No Ct	No Ct	No Ct	No Ct	No Ct
114	Contact	No Ct	No Ct	No Ct	No Ct	No Ct	No Ct	No Ct	No Ct	No Ct	No Ct	No Ct
120	Inoc.	No Ct	No Ct	No Ct	No Ct	No Ct	No Ct	No Ct	No Ct	No Ct	No Ct	No Ct
121	Contact.	No Ct	No Ct	No Ct	No Ct	No Ct	No Ct	No Ct	No Ct	No Ct	No Ct	No Ct
124	Inoc.	No Ct	No Ct	No Ct	No Ct	No Ct	No Ct	No Ct	No Ct	No Ct	No Ct	No Ct
125	Contact.	No Ct	No Ct	No Ct	No Ct	No Ct	No Ct	No Ct	No Ct	No Ct	No Ct	No Ct
111	Inoc.	No Ct	No Ct	No Ct	No Ct	No Ct	No Ct	No Ct	No Ct	No Ct	No Ct	No Ct
109	Contact	No Ct	No Ct	No Ct	No Ct	No Ct	No Ct	No Ct	No Ct	No Ct	No Ct	No Ct
123	Inoc.	No Ct	No Ct	No Ct	No Ct	No Ct	No Ct	No Ct	No Ct	No Ct	No Ct	No Ct
122	Contact	No Ct	No Ct	No Ct	No Ct	No Ct	No Ct	No Ct	No Ct	No Ct	No Ct	No Ct
127	Inoc.	No Ct	No Ct	No Ct	No Ct	x	x	x	x	x	x	x
128	Contact	No Ct	No Ct	No Ct	No Ct	x	x	x	x	x	x	x
129	Inoc.	No Ct	No Ct	No Ct	No Ct	No Ct	No Ct	No Ct	No Ct	No Ct	No Ct	No Ct

a Inoc, bat oronasally inoculated with 10^5^TCID_50_ SARS-CoV-2; Contact, uninoculated bat cohoused with inoculated bat to determine virus transmission.

b Day postinoculation.

c No Ct, no detectable real-time PCR amplification. Quantities of virus based on standard curve of known virus concentrations and expressed as TCID_50_ equivalents/mL.

d x, Bat euthanized after previous sampling DPI.

An oral swab from a positive bat (bat 118 DPI 8) was inoculated into Vero E6 tissue culture to verify viral viability. This resulted in positive virus isolates from the first and third passages that were confirmed to be SARS-CoV-2 by qRT-PCR analyses as described above. Whole-genome sequencing of these isolates revealed that they shared the same genetic changes as the WA-1 isolate used as inoculum, compared to the original Wuhan SARS-CoV-2 isolate. Two additional, consistent changes in both passages of the bat 118 DPI 8 isolate were found that were not in the WA-1 inoculum isolate. These are shown in Table S2. Both changes were in open reading frame 8 and were synonymous (F > F).

10.1128/msphere.00263-22.2 TABLE S2 Variant detection in SARS-CoV-2 isolated from Bat 118, DPI 8. Genetic changes in the SARS-CoV-2 inoculum strain (WA1) and Bat 118 DPI 8 after one passage in Vero cells (GenBank OM995890) compared with the reference strain (Wuhan HU-1; GenBank MN908947). No additional genetic changes were observed after two additional passages in Vero cells (GenBank OM995891). Sample ID, sample name. Reference Position, location of genetic variant on Wuhan HU-1 genome. Reference: nucleotide sequence in HU-1. Variant: nucleotide sequence in WA-1 and bat isolate. AA Change: potential change in amino acid coding genes affected by variant. Download Table S2, DOCX file, 0.01 MB. Copyright © 2023 Hall et al. 2023 Hall et al. https://creativecommons.org/licenses/by/4.0/ This is an open-access article distributed under the terms of the Creative Commons Attribution 4.0 International license.

### Serological analyses.

SARS-CoV-2 antibodies were detected by competitive enzyme-linked immunosorbent assay (cELISA) in five T. brasiliensis sera (DPI 20) with percent inhibitions ranging from about 34 to 79% (Table 3). These five bats were the same five that were orally excreting virus detected by qRT-PCR (Table 1). These seropositive bat sera were subsequently tested at dilutions from 1:20 to 1:80. One serum (110) was positive only at the original 1:10 dilution. Two sera (123, 124) were positive at 1:20, whereas two sera (104, 118) remained positive at 1:40 dilutions. All of the remaining bats remained seronegative by this assay.

**TABLE 3:** Competitive ELISA^*a*^ results of Mexican free-tailed bat sera tested after experimental challenge with SARS-CoV-2

		Results^*c*^			
Bat ID	%Inhibition^*b*^	1:10	1:20	1:40	1:80
102^*d*^	−2.656	Neg			
103	14.73	Neg			
104	62.75	**Pos**	**Pos**	**Pos**	Neg
105^*d*^	−157.1	Neg			
108^*d*^	12.66	Neg			
109	13.49	Neg			
110	34.32	**Pos**	Neg	Neg	Neg
111	1.690	Neg			
112	25.28	Neg			
113	15.21	Neg			
114	−34.25	Neg			
117	17.42	Neg			
118	79.30	**Pos**	**Pos**	**Pos**	Neg
119^*e*^	14.45	Neg			
120	0.862	Neg			
121	14.25	Neg			
122	−24.39	Neg			
123	65.44	**Pos**	**Pos**	Neg	Neg
124	57.30	**Pos**	**Pos**	Neg	Neg
125	−31.63	Neg			
127	−15.07	Neg			
128	28.04	Neg			
129	7.899	Neg			

a SARS-CoV-2 sVNT, GenScript, Piscataway, NJ.

b Inhibition of >30% is positive. Positive results in bold. Percent inhibitions are presented for the 1:10 dilutions of sera.

c Twofold serial dilutions of positive sera.

d Uninoculated controls.

e Bats euthanized prior to the study.

### Clinical signs of infection, postmortem examination, and histopathology.

Over the 3-week course of this study, no overt clinical signs of SARS-CoV-2 disease were observed in 18/21 bats, including the five bats that became infected and were orally excreting virus. These bats maintained or gained weight during the study (Table 4) and appeared healthy. One inoculated bat (127) presented with lethargy, decreased respiratory rate, and increased respiratory effort; and was euthanized on DPI 6, along with its cagemate (128). An additional bat (112) presented with lethargy, obtundation, weight loss, hypersalivation, inability to swallow, and respiratory distress and was euthanized at DPI 10.

**TABLE 4:** Body weights (g) of SARS-CoV-2 inoculated Mexican free-tailed bats

Bat ID	Treatment	DPI^*a*^ 0	DPI 2	DPI 4	DPI 6	DPI 8	DPI 10	DPI 12	DPI 14	DPI 16	DPI 18	DPI 20
102	Control	10.19	10.11	11.23	11.27	11.8	12.13	11.78	11.7	13.45	13.13	13.24
108	Control	11.97	11.42	12.1	11.86	12.34	12.48	12.3	12.93	12.31	14.38	14.46
104	Inoculated	11.99	10.56	11.85	12.13	12.9	12.76	12.81	13.11	X^*b*^	x	x
103	Contact	11.81	10.31	11.3	11.28	11.56	12.12	11.88	12.81	x	x	x
112	Inoculated	11.75	11.97	11.31	9.72	9.26	8.4	x	x	X	x	x
113	Contact	14.37	13.25	12.84	13.81	14.13	15.08	14.83	15.01	14.67	14.43	14.43
110	Inoculated	16.19	14.34	15.39	15.14	15.36	15.6	16.03	15.09	16.04	16.46	16.66
117	Contact	12.45	11.26	11.84	11.53	11.83	12.87	12.86	12.18	12.66	13.09	13.13
118	Inoculated	13.45	12.34	12.87	12.56	12.9	13.57	13.7	13.72	13.84	13.91	13.75
114	Contact	10.85	10.2	10.92	10.66	11.31	11.61	11.7	11.39	11.29	11.27	13.68
120	Inoculated	12.1	10.73	11.71	11.5	11.63	12.14	12.27	12.48	12.9	12.47	12.37
121	Contact	10.33	11.1	12	11.53	11.55	12.05	11.97	12.04	12.54	12.49	13.11
124	Inoculated	13.25	11.95	12.87	12.52	13.33	13.73	14.05	14.49	14.44	14.09	14.9
125	Contact	13.07	12.2	12.45	12.16	11.57	12.05	12.19	12.41	12.36	12.56	12.84
111	Inoculated	14.02	13.11	13.15	13.73	13.62	13.92	14.25	13.95	13.98	13.64	13.93
109	Contact	10.86	11.29	11.14	10.56	10.64	10.99	11.39	12.05	12.14	11.97	12.23
123	Inoculated	11.09	10.45	10.83	10.31	10.62	11.19	11.5	11.78	11.48	11.84	12.12
122	Contact	9.23	9.45	9.78	10.33	10.6	11.37	11.72	11.57	11.65	11.53	11.71
127	Inoculated	9.13	8.86	9.37	8.69	x	x	x	x	X	x	x
128	Contact	8.39	8.12	8.07	7.95	x	x	x	x	X	x	x
129	Inoculated	10.4	10.96	10	11.51	10.92	10.7	11.48	12.02	10.74	10.76	11.39

a Day postinoculation.

b x, Bat euthanized after previous sampling DPI.

Upon further examination, the bat that presented with respiratory difficulty (bat 127) and its cagemate (128) were in poor or emaciated body condition, respectively, with minimal or no fat stores. Bat 112, euthanized due to respiratory distress and hypersalivation, was in fair body condition. All other bats were in good body condition evidenced by moderate to abundant fat stores.

Our histopathologic findings for all examined bats, including the five bats that were infected and recovered, were consistent with the euthanasia procedures utilized, parasitism, or bacterial infections and were not consistent with findings observed in other animals experimentally infected with SARS-CoV-2 (12–14).

### Immunohistochemistry for detection of SARS-CoV-2 antigen.

Sections from the rostral nasal cavity, lung, heart, spleen, liver, pancreas, stomach, small and large intestine, and brain from a total of 14 experimentally and mock inoculated bats were subjected to immunohistochemistry for the detection of SARS-CoV-2 antigen. No viral antigen was detected in any of the tissues examined from these bats (Table S3).

10.1128/msphere.00263-22.3 TABLE S3 SARS-CoV-2 antigen distribution in selected tissues from SARS-CoV-2 inoculated and uninoculated control Mexican free-tailed bats. Bats euthanized, necropsied and tissue samples collected on day postinoculation (DPI) 7 (bats 127, 128); DPI 10 (bat 112); DPI 14 (bats 103, 104); and DPI 20 for the remaining bats. ^1^Uninoculated control. ^2^SARS-CoV-2-infected bats. None: no detectable SARS-CoV-2 nucleocapsid antigen. Download Table S3, DOCX file, 0.01 MB. Copyright © 2023 Hall et al. 2023 Hall et al. https://creativecommons.org/licenses/by/4.0/ This is an open-access article distributed under the terms of the Creative Commons Attribution 4.0 International license.

### SARS-CoV-2 in bat tissues.

qRT-PCR analyses of tissues collected from the infected and control bats did not detect viral RNA in any tissue from any of the bats examined (Table 5).

**TABLE 5:** qRT-PCR analyses of selected tissues from SARS-CoV-2 infected and uninoculated control Mexican free-tailed bats^*a*^

	Bat ID						
Tissue	102^*b*^	108^*b*^	104	110	118	123	124
Brain	NoCt^*c*^	NoCt	NoCt	NoCt	NoCt	NoCt	NoCt
Nares	NoCt	NoCt	NoCt	NoCt	NoCt	NoCt	NoCt
Lung cranial	NoCt	NoCt	NoCt	NoCt	NoCt	NoCt	NoCt
Lung, caudal	NoCt	NoCt	NoCt	NoCt	NoCt	NoCt	NoCt
Heart	NoCt	NoCt	NoCt	NoCt	NoCt	NoCt	NoCt
Liver	NoCt	NoCt	NoCt	NoCt	NoCt	NoCt	NoCt
Kidney	NoCt	NoCt	NoCt	NoCt	NoCt	NoCt	NoCt
Spleen	NoCt	NoCt	NoCt	NoCt	NoCt	NoCt	NoCt
Small intestine	NoCt	NoCt	NoCt	NoCt	NoCt	NoCt	NoCt
Colon	NoCt	NoCt	NoCt	NoCt	NoCt	NoCt	NoCt

a Tissues collected were from bats euthanized and necropsied on DPI 20.

b Uninoculated control.

c NoCt, no detectable RT-PCR.

## DISCUSSION

We experimentally challenged Mexican free-tailed bats with SARS-CoV-2. Of the 10 inoculated bats, 5 orally excreted virus for 6 to 18 days postchallenge. These five also mounted an immune response and cleared the virus before the end of the study. Based on these findings, we concluded that Mexican free-tailed bats are susceptible to infection by SARS-CoV-2.

We found no evidence of transmission between cohoused T. brasiliensis. Five of the 10 (50%) inoculated bats became infected indicating that our inoculum titer (10^5^ TCID_50/_dose) was apparently near the 50% infectious dose for this species. However, the largest amount of virus excreted by infected bats was between 10^3^ and 10^4^ TCID_50_ equivalents/mL. Thus, based on these data, contact transmission between T. brasiliensis would be unlikely.

Another possible reason for no transmission between T. brasiliensis was the way we housed the bats during the challenge study. In the wild, T. brasiliensis roost in very dense colonies that aggregate in large numbers in natural and anthropogenic structures such as bridges, caves, and culverts. In our study, we cohoused two bats, one inoculated and one uninoculated, in cages of approximately 1 meter^3^. This relatively large space did not force the bats to congregate as closely as occurs in natural roosts and thus “social distancing” may have impeded viral transmission. In future challenge studies, we plan to adjust the bat housing to help take this factor into account.

In addition to the apparent lack of transmission, another observation from this study is that the virus was not excreted via the digestive system. Given the sizes of T. brasiliensis colonies, large amounts of fecal material accumulate that could potentially become a source of infectious virus. Other coronaviruses are typically found in the digestive tracts of bats; however, surprisingly, we found no evidence of virus in the digestive tracts or in rectal swabs of any bat, including in the 5 infected bats.

Another finding was that T. brasiliensis showed no obvious adverse health effects from SARS-CoV-2 infection. While it is unknown if infected wild bats would have a diminished capacity to forage for food, perform maternal care, or other life functions, our findings indicate that T. brasiliensis populations are likely not at risk from the pandemic.

Regardless, an accurate determination of the infectious dose of SARS-CoV-2 in T. brasiliensis would be an important next study. We do not know if the amounts of virus excreted are enough to infect other mammalian species, including humans, or if T. brasiliensis can be infected with SARS-CoV-2 by exposure to sick humans. However, the dose needed to infect humans is between 10^1^ and 10^2^ TCID_50_, so people that contact infected T. brasiliensis would be at risk of acquiring an infection from an infected bat. Because this species often resides in urban settings, these are important public health and pandemic ecological concerns.

We were unable to conduct necropsies and collect tissues from actively infected animals. All bats euthanized for these purposes during the study were not among the infected cohort, and by the end of the study, all infected bats had cleared the virus. Therefore, the qRT-PCR, pathology, and histochemistry results from bat tissues were inconclusive.

It is generally accepted that SARS-CoV-2 originated in wild horseshoe bats (*Rhinolophus* sp.) from China, subsequently transmitted to other host species, and ultimately infected humans, leading to a pandemic (6). The virus also infects at least one other bat species, Egyptian fruit bats, Rousettus aegyptiacus (13). This has raised concerns that the virus could infect North American bats, some of whose populations are under severe pressure from other diseases and from habitat degradation. Hall et al. (11) previously showed that big brown bats, a species commonly encountered by humans, are resistant to infection by SARS-CoV-2. In this study, however, we demonstrated that T. brasiliensis, a migratory bat that resides in large colonies, often in urban areas, is susceptible to infection by this virus. Based on the comparative structure of the SARS-CoV-2 cellular receptor, Damas et al. (15) predicted that T. brasiliensis were unlikely to become infected with SARS-CoV-2. However, other researchers have shown that the T. brasiliensis receptor does permit infection by SARS-CoV-2 (16). Thus, it is likely that other factors are involved in mediating susceptibility to this virus after cellular entry. The genetic changes we detected in viral isolates from the swab of one infected bat indicate that mutations can occur rapidly in a new host and that genetic analyses of recovered viral isolates may further inform our understanding of the emergence of novel viral variants. Our findings also indicate that the susceptibility of each species to SARS-CoV-2 is independent and each species would benefit from being examined individually. These results have implications for bat rehabilitators, wildlife biologists, cave recreationists, and the public at large, if they come into contact with Mexican free-tailed bats or enter caves or other environments where bats are roosting.

Supporting data are available at https://doi.org/10.5066/P9RDA1H6 (17).

## MATERIALS AND METHODS

### Virus acquisition and propagation.

We obtained the SARS-CoV-2 virus (2019-nCoV/USA-WA1/2020) from BEI Resources, National Institute of Allergy and Infectious Diseases (NIAID), National Institutes of Health (NIH) (Manassas, VA). The virus was isolated from the first confirmed patient with coronavirus disease 2019 (COVID-19) in the United States (18). We propagated and quantified the virus in Vero E6 cell culture using standard techniques (virus isolate provided by BEI Resources [Manassas, VA] with one additional passage in cell culture).

### Animal acquisition and husbandry.

All husbandry and experimental protocols were approved by the U.S. Geological Survey (USGS) National Wildlife Health Center (NWHC) Institutional Animal Care and Use Committee. Wild T. brasiliensis were captured in Williamson County, Texas in August 2021. A mixture of adult and juvenile male bats was collected and immediately placed into a temperature-controlled chamber maintained at approximately 20°C. This temperature induced the bats to enter torpor during transport to the NWHC (Madison, WI).

On arrival at the NWHC, the bats were given veterinary examinations and treated topically with selamectin for parasites (Zoetis, Florham Park, NJ). The bats were hand fed mealworms (Tenebrio molito) supplemented with a vitamin and mineral mixture, and water was provided *ad libitum.* Bats underwent a quarantine and acclimatization period of 30 days prior to commencement of this study during which time the bats learned to feed themselves.

### Preinoculation fecal sampling and coronavirus analysis.

During the acclimatization period, we collected fecal samples from the individual bats to determine the presence of other coronaviruses in these subjects. Each fecal sample was suspended 10% (wt/vol) in viral transport medium (VTM; Hank’s balanced salt solution, 0.05% gelatin, 5% glycerin, 1,500 units/mL penicillin, 1,500 mg/mL streptomycin, 0.1 mg/mL gentamicin, and 1 mg/mL fungizone). Viral RNA was extracted using the MagMax Pathogen RNA/DNA kit (Applied Biosystems, Forest City, California) on a Kingfisher Flex magnetic particle processor according to the manufacturer's instructions. The presence of coronaviruses was determined using methods previously described (19).

### Virus inoculation.

Experimental inoculations were performed under Biosafety Level-3 conditions at the NWHC. We utilized 21 male Mexican free-tailed bats after the acclimatization period, and pairs of bats were cohoused in mesh cages. One bat from each of nine bat pairs was inoculated with SARS-CoV-2, and its cagemate was left uninoculated to determine if the virus could be horizontally transmitted between bats. One bat was inoculated and housed individually. The SARS-CoV-2 inoculum dose was 10^5^ TCID_50/_bat and was administered nasally (4 μL) and orally (6 μL) using a micropipette. One bat pair was sham inoculated with the same volume of VTM. This technique has been used to inoculate other species with SARS-CoV-2 (11–14). The inoculum titer was verified by qRT-PCR as described below and virus viability confirmed in cell culture using Vero E6 cells.

### Animal monitoring and sampling.

Bats were observed at least twice daily to monitor health status and document development of clinical signs. Just prior to inoculation and every other day thereafter, each bat was weighed, and oropharyngeal and rectal swabs (Puritan Medical Products, Guilford, ME) were collected and placed in 0.5 mL VTM. On DPI 7 and on DPI 14, bats from one cage (one inoculated bat, one uninoculated) were euthanized by an overdose of isoflourane, a postmortem examination was conducted, and tissues (nares, caudal lung, cranial lung, heart, liver, spleen, kidney, small intestine, colon, and brain) and blood collected. At the end of the study (DPI 20), all remaining bats were euthanized and postmortem examinations were completed for the control bats and an additional cage pair. Blood was collected for serological analyses from all euthanized bats.

### qRT-PCR analyses.

RNA extractions of swab material were performed in 96-well plates using Mag Max-96 AI/ND Viral RNA isolation kit (Applied Biosystems, Foster City, CA) following the manufacturer’s instructions. A positive-control sample consisting of a 1:100 dilution of the SARS-CoV-2 inoculum used in the study was included with each extraction series to validate successful RNA extraction. qRT-PCR analyses were conducted utilizing the Centers for Disease Control 2019-nCoV N1 primers and probe (https://www.cdc.gov/coronavirus/2019-ncov/lab/rt-pcr-panel-primer-probes.html) and AgPath-ID one-step RT-PCR reagents (Ambion/ThermoFisher, Waltham, MA). We included a standard curve of serial dilutions of RNA extracted from SARS-CoV-2 virus stock (10^7^ TCID_50_/mL) in each qRT-PCR assay to quantify viral amounts.

### Rabies diagnostics.

Brain tissue was assessed for rabies infection using DFA. After brain impressions were fixed in acetone, slides were stained with a FITC-labeled monoclonal antibody (mAB) conjugate (Fujirebio U.S. Inc., Malvern, PA, USA) and visualized under a fluorescence microscope (20).

### Necropsy and histopathology.

Two animals (inoculated and uninoculated cagemates) were euthanized at DPI 7 (bats 127 and 128) and DPI 14 (bats 103 and 104) and an additional 2 sets of cagemates at DPI 20 (bats 109, 110, 111, and 117), using an overdose of isoflourane with subsequent decapitation. Two uninoculated control animals (102 and 108) were also euthanized at DPI 20. These subjects were immediately necropsied after euthanasia and body condition and gross observations were recorded. Tissues were collected as described above and saved frozen at –80°C for virological analyses. Additional tissue portions were fixed in 10% neutral buffered formalin for histological analysis. For histopathological examination, fixed tissues were processed routinely, sectioned at approximately 5 μm and stained with hematoxylin and eosin at the Wisconsin Veterinary Diagnostic Laboratory (Madison, WI). At DPI 20, all remaining bats were euthanized, serum was collected, and all bat carcasses were saved frozen. Three bats (118, 123, and 124) that were saved frozen and later shown to be infected by swab analysis were subsequently necropsied and sampled.

### SARS-CoV-2-specific immunohistochemistry.

For immunohistochemistry (IHC), 4-μm sections of formalin-fixed paraffin-embedded tissue were mounted on positively charged Superfrost Plus slides and subjected to IHC using an anti-nucleocapsid rabbit monoclonal antibody (HL344, Cell Signaling Technology, Danvers, MA). IHC was performed using the automated BOND-RXm platform and the Polymer Refine Red Detection kit (Leica Biosystems, Wetzlar, Germany). Following automated deparaffinization, heat-induced epitope retrieval was performed using a ready-to-use citrate-based solution (pH 6.0; Leica Biosystems) at 100°C for 20 min. Sections were then incubated with the primary antibody (diluted at 1:1,600 in primary antibody diluent [Leica Biosystems]) for 30 min at room temperature, followed by a polymer-labeled goat anti-rabbit IgG coupled with alkaline phosphatase (30 min). Fast Red was used as the chromogen (15 min), and counterstaining was performed with hematoxylin for 5 min. Slides were dried in a 60°C oven for 30 min and mounted with a permanent mounting medium (Micromount, Leica Biosystems). Lung sections from a SARS-CoV-2-infected hamster were used as positive assay controls.

### Virus RNA extraction and qRT-PCR from bat tissues.

Approximately 10 mg of each tissue was macerated in extraction buffer and RNA extracted using the ZYMO Research Quick DNA/RNA Pathogen Miniprep kit (ZYMO Research, Irvine, CA) according to the manufacturer’s directions. qRT-PCR analyses were performed as described above.

### Antibody detection.

To detect neutralizing antibodies to SARS-CoV-2, bat sera were screened at a 1:10 dilution using a competitive enzyme linked immunosorbent assay (SARS-CoV-2 sVNT; GenScript, Piscataway, NJ) according to the manufacturer’s instructions. As directed, a reduction in optical density (OD) of ≥30% compared to the mean OD of the negative control was considered positive for the presence of neutralizing antibodies. In addition to the positive control provided in the kit, we used positive guinea pig serum obtained through BEI Resources, NIAID, NIH: Polyclonal Anti-SARS Coronavirus antiserum (Guinea Pig, NR-10361). To determine neutralizing antibody titers from positive sera, samples were 2-fold serially diluted and titers recorded as the reciprocal of the endpoint dilution where the serum was no longer considered positive.

### Virus recovery and whole-genome sequencing.

Oral swab VTM from Bat 118 DPI 8 was inoculated into Vero E6 cells and incubated at 37°C, 5% CO_2_ for 7 days. The flasks were examined daily for cytopathic effects. Cell lysates collected after three cycles of freezing and thawing were used for RNA extraction and serial passage. Extracted RNA was converted to cDNA with SuperScript IV (ThermoFisher, Waltham, MA) or Maxima H minus (ThermoFisher, Waltham, MA) reverse transcriptase according to manufacturer’s instructions. Tiled amplicon sequencing by the ARTIC method (21) was performed using Oxford Nanopore Technology’s MinION running on a MK1C instrument. Bioinformatic analysis was performed using the CLC Genomics Workbench v22 (Qiagen, Redwood City, CA) using a publicly available workflow (https://storage.googleapis.com/theiagen-resources/qiagen/SARS-CoV-2_Tutorial.zip).
