# Supplementary material for: Experimental Infection of Mexican Free-Tailed Bats (Tadarida brasiliensis) with SARS-CoV-2
Source: mSphere. 2023 Jan 4;8(1):e00263-22. doi: 10.1128/msphere.00263-22 (PMC9942575; doi:10.1128/msphere.00263-22)
Supplement: TABLE S1 [file msphere.00263-22-s0001.docx]

| **Bat ID** | **Age^1^** | **Treatment** | **DFA**^2^ | **Coronavirus** |
| --- | --- | --- | --- | --- |
| 101 | j | None^3^ | Pos | Neg |
| 105 | j | None^3^ | Neg | Neg |
| 107 | j | None^3^ | Neg | Neg |
| 115 | j | None^3^ | Neg | Neg |
| 119 | j | None^3^ | Neg | Neg |
| 102 | j | Control | Neg | Neg |
| 108 | j | Control | Neg | Neg |
| 104 | a | Inoculated | Neg | Neg |
| 103 | j | Contact | Neg | Neg |
| 112 | j | Inoculated | Neg | Neg |
| 113 | a | Contact | Neg | Neg |
| 110 | a | Inoculated | Neg | Neg |
| 117 | a | Contact | Neg | Neg |
| 118 | a | Inoculated | Neg | Neg |
| 114 | j | Contact | Neg | Neg |
| 120 | j | Inoculated | Neg | Neg |
| 121 | j | Contact | Neg | Neg |
| 124 | j | Inoculated | Neg | Neg |
| 125 | a | Contact | Neg | Neg |
| 111 | j | Inoculated | Neg | Neg |
| 109 | j | Contact | Neg | Neg |
| 123 | a | Inoculated | Neg | Neg |
| 122 | j | Contact | Neg | Neg |
| 127 | a | Inoculated | Neg | Neg |
| 128 | a | Transmission | Neg | Neg |
| 129 | j | Inoculated | Neg | Neg |
