# Supplementary material for: Experimental Infection of Mexican Free-Tailed Bats (Tadarida brasiliensis) with SARS-CoV-2
Source: mSphere. 2023 Jan 4;8(1):e00263-22. doi: 10.1128/msphere.00263-22 (PMC9942575; doi:10.1128/msphere.00263-22)
Supplement: TABLE S2 [file msphere.00263-22-s0002.docx]

Sample ID Reference Position Reference Sequence Variant AA Change

WA-1 8782 C T ORF1ab AGC > AGT (Ser > Ser)

28144 T C ORF8 TTA > TCA (Leu > Ser)

Bat 118 DPI 8 8782 C T ORF1ab AGC > AGT (Ser > Ser)

28144 T C ORF8 TTA > TCA (Leu > Ser)

28253 C T ORF8 TTC > TTT (Phe > Phe)

28603 C T N TTC > TTT (Phe > Phe
