# Supplementary material for: Experimental Infection of Mexican Free-Tailed Bats (Tadarida brasiliensis) with SARS-CoV-2
Source: mSphere. 2023 Jan 4;8(1):e00263-22. doi: 10.1128/msphere.00263-22 (PMC9942575; doi:10.1128/msphere.00263-22)
Supplement: TABLE S3 [file msphere.00263-22-s0003.docx]

| Tissue | 102^1^ | 108^1^ | 103 | 104^2^ | 109 | 110^2^ | 111 | 112 | 117 | 118^2^ | 123^2^ | 124^2^ | 127 | 128 |  |
| --- | --- | --- | --- | --- | --- | --- | --- | --- | --- | --- | --- | --- | --- | --- | --- |
|  |  |  |  |  |  |  |  |  |  |  |  |  |  |  |  |
| Brain | None | None | None | None | None | None | None | None | None | None | None | None | None | None |  |
| Nares | None | None | None | None | None | None | None | None | None | None | None | None | None | None |  |
| Lung | None | None | None | None | None | None | None | None | None | None | None | None | None | None |  |
| Heart | None | None | None | None | None | None | None | None | None | None | None | None | None | None |  |
| Liver | None | None | None | None | None | None | None | None | None | None | None | None | None | None |  |
| Kidney | None | None | None | None | None | None | None | None | None | None | None | None | None | None |  |
| Spleen | None | None | None | None | None | None | None | None | None | None | None | None | None | None |  |
| Stomach | None | None | None | None | None | None | None | None | None | None | None | None | None | None |  |
| Pancreas | None | None | None | None | None | None | None | None | None | None | None | None | None | None |  |
| Small intestine | None | None | None | None | None | None | None | None | None | None | None | None | None | None |  |
| Colon | None | None | None | None | None | None | None | None | None | None | None | None | None | None |  |
